# Perceived Ownership of Avatars Influences Visual Perspective Taking

**DOI:** 10.3389/fpsyg.2018.00743

**Published:** 2018-05-25

**Authors:** Christian Böffel, Jochen Müsseler

**Affiliations:** Institute of Psychology, RWTH Aachen University, Aachen, Germany

**Keywords:** avatars, ownership, stimulus-response compatibility tasks (SRC), perspective-taking, human computer interaction (HCI)

## Abstract

Modern computer-based applications often require the user to interact with avatars. Depending on the task at hand, spatial dissociation between the orientations of the user and the avatars might arise. As a consequence, the user has to adopt the avatar’s perspective and identify herself/himself with the avatar, possibly changing the user’s self-representation in the process. The present study aims to identify the conditions that benefit this change of perspective with objective performance measures and subjective self-estimations by integrating the idea of avatar-ownership into the cognitive phenomenon of spatial compatibility. Two different instructions were used to manipulate a user’s perceived ownership of an avatar in otherwise identical situations. Users with the high-ownership instruction reported higher levels of perceived ownership of the avatar and showed larger spatial compatibility effects from the avatar’s point of view in comparison to the low ownership instruction. This supports the hypothesis that perceived ownership benefits perspective taking.

## Introduction

When we are confronted with avatars in the virtual world, we often have to adopt their perspective in order to complete our task. Sometimes it is necessary to control an avatar; sometimes we merely interact with avatars controlled by others. Avatars are used to represent the user in the digital world and the user is able to interact with the virtual world through the avatar. In both situations, seeing the world through the avatar’s eyes can be useful to plan actions or to interpret the actions of others. This process, referred to as visual perspective taking (PT), was observed in various situations and toward a large variety of targets. It occurs toward human confederates (Frischen et al., 2009; Freundlieb et al., 2016, 2017) and even non-human targets like triangles (Zwickel, 2009) or arrows (Santiesteban et al., 2014). In the case of avatars, the distinction between an object and a person is not clear-cut. It is sometimes unclear if someone else controls the avatar or if the avatar is an independent agent controlled by the program itself. While we are generally able to identify objects as non-human, we still tend to attribute human-like agency and mental states to them (Heider and Simmel, 1944). This agency attribution seems to aide visual PT (Zwickel, 2009). In past studies we showed that PT occurs toward avatars, regardless of whether PT is needed to complete the task (Müsseler et al., 2017) or not (Böffel and Müsseler, 2017). In the present study we confronted participants with an avatar that was presented opposite to them on a computer screen in a top–down view. Our goal was to take a closer look at how this avatar is interpreted and how this interpretation can benefit or inhibit PT in a top–down manner.

People have the remarkable ability to incorporate objects — like avatars — into the mental representation of their bodies. The most famous example of this is the rubber hand illusion experiment by Botvinick and Cohen (1998) in which participants were able to feel the touch on a rubber hand. To achieve this illusion, a rubber hand was placed in front of the participants while their real hand was hidden from sight. When the rubber hand and the real hand were brushed synchronously, participants started to “feel” the stimulation on the rubber hand instead of their own and reported that the rubber hand seemed to be part of their body. This sense that an object belongs to the person’s own body is referred to as *ownership*. Ma and Hommel (2013) demonstrated that virtual objects — such as virtual hands — could also become part of mental body representations akin to the rubber hand.

Several factors were identified that lead to this sense of ownership. Makin et al. (2008) showed the importance of spatial congruency between the real and the artificial hand for perceived ownership, whereas Shimada et al. (2009) demonstrated the necessity of temporal congruency between the tactile stimulation and the visual perception of this stimulation on the fake hand. Tsakiris (2010) underlined the importance of visual similarity between real and artificial body parts and argued that the artificial body part has to resemble the real body part in order to be embodied. However, this assumption has been called into question by recent experimental findings. Armel and Ramachandran (2003) were able to observe ownership of a table, Ma and Hommel (2015a) demonstrated ownership of objects like balloons and squares and Guterstam et al. (2013) were even able to observe embodiment of empty space. These effects are overall comparable to the perceived ownership of artificial hands even though the objects had no resemblance of real hands or body parts.

A different factor that seems to influence ownership is a sense of connectedness between the actions of the person and the action effects on the side of the object. This controlling aspect — or perceived agency over the object — leads to ownership (Ma and Hommel, 2015a,b) and it can be used to induce a rubber hand-like illusion without the need for tactile stimulation (Kalckert and Ehrsson, 2014). Overall, perceived agency over an object is a promising mechanism for inducing ownership that also influences visual PT. When comparing PT in situations in which a person was actually controlling the arms of an avatar to situations in which this control was merely imagined, only the conditions with actual control were associated with visual PT (Böffel and Müsseler, 2017). Combined with the results of Zwickel (2009), two conflicting characteristics of a situation can be identified that seem to benefit PT: on the one hand perceiving the target as an individual agent seems to aide PT, because it can help us understand someone’s actions (Tversky and Hard, 2009), on the other hand, actual control over the target also seems to lead to PT. Both processes seem exclusive, because we cannot see an avatar as an independent agent and attribute intentions to it, if it fully obeys our every command. Our goal is to solve this conflict in the present study.

The results of previously mentioned studies point toward the importance of bottom–up processes in the integration of objects into a person’s representation of their action and body. It therefore seems plausible that action representation is a gateway that leads to ownership: if a person’s action reliably causes a certain effect, even when this effect is produced through seemingly unconnected mediating objects (e.g., a rubber hand or a balloon), the action effect becomes a relevant part of the action code and the person is able to anticipate this effect as a consequence of her/his action. We believe that ownership of the object that produces the action effect is inferred as a result. We expect that once ownership is acquired, visual PT often follows to facilitate the planning of future actions through the object that is now perceived as part of the person’s body.

Because ownership is observed in different situations and of different objects, it seems very likely that ownership toward an avatar is rather easily achievable, especially if the person controls the avatar. Although past studies point to the importance of bottom–up processes for ownership, we aim to demonstrate that it is also subject to top–down modulation. The acquisition of ownership via bottom–up processes seems to be an automatic process and top–down modulation of automatic processes has been demonstrated before (for an overview see Kiefer, 2007). We believe that when confronted with an object, two different explanations of the same situation are able to alter the framework that influences how ownership of an object is acquired. In a high ownership explanation, the participants might shift attention to situational features that support their sense of control, while in the low ownership explanation the opposite is expected. The use of two different instructions that target this sense of control should therefore be able to alter the interpretation of the situation resulting in different levels of perceived ownership of an avatar (measured via self-report questionnaire). Such a result could further our understanding of the nature and dependencies of automatic processes.

When examining visual PT, two different — although similar — tasks have been used in the past: the *own body transformation task* that asks participants to judge on which side of a shown body a certain salient feature is located, and the *avatar-in-scene task* that uses laterality decisions of objects from an avatars point of view. Both tasks share the problem that the results are potentially influenced by stimulus–response compatibility (SRC) effects with unknown consequences (May and Wendt, 2013). SRC refers to the observation that certain mappings of responses to stimuli lead to performance advantages over others (Fitts and Deininger, 1954) and result in faster reaction times and lower error rates. When using stimuli and responses that carry spatial information, SRC is in most cases aligned with spatial correspondence of stimulus and response positions. Conditions in which stimulus and response occur in the same hemifield are generally compatible, whereas conditions with opposing positions are incompatible (for an overview see Proctor and Vu, 2006). A theoretical framework often used to explain SR compatibility are the so called dual-route models (e.g., Kornblum et al., 1990). These models propose that stimulus presentation causes the activation of two routes: the automatic route leads to a direct activation of a response code that spatially corresponds to the stimulus position. A stimulus presented on the left would activate a left response code. A second route uses the SR mapping, for example given by an instruction, to retrieve the correct response. In the case that both routes activate the same correct response the execution of the response is facilitated, otherwise a conflict occurs that has to be resolved. This conflict leads to slower reaction times and increased error rates. As a consequence of SRC, both, facilitation and interference can be observed (Wallace, 1971). Because SRC effects are often attributed to an overlap of certain features of the task’s mental representation, we can use these effects to infer how a certain stimulus is mentally represented. More importantly, it allows us to identify if the stimulus position is coded from the participants own point of view, leading to the typically observed advantages of spatially corresponding stimulus–response parings, or if it produces different compatibility effects indicating that the stimulus position is coded from the avatar’s point of view instead. The results of this coding process — referred to as feature codes — are often seen as abstract representations and independent of the modality that was used to create them (Hommel et al., 2001). As consequence, a stimulus coded as “left” from the participant’ point of view would form the same feature code as a stimulus coded as “left” from an Avatar’s point of view, although they do in fact occupy different locations.

In the present study we use SRC effects to measure visual PT (Böffel and Müsseler, 2017; Müsseler et al., 2017). Previous studies that follow a similar approach point toward a complicated situation. On one hand, studies find that objects are generally coded from the person’s own perspective when no agency instruction is used (Gardner and Potts, 2011; Taylor et al., 2016). However, this observation doesn’t seem universal. Gardner and Potts (2010) and Taylor et al. (2016) show that under certain circumstances feature codes can be created from the objects perspective instead. Müsseler et al. (2017) demonstrated that SRC effects can arise from an avatar’s point of view, rather than the person’s own in situations that force the person to take the avatar’s perspective in a SRC task and Böffel and Müsseler (2017) showed that these effects can occur even in a Simon task, in which the avatar’s orientation is irrelevant. However, these compatibility changes only occur when the dimensional overlap between stimulus and response position from the participant’s point of view is low, or the participant’s control over the avatar’s movements is high. The latter is likely linked to ownership of the avatar that is acquired through bottom–up processes and effect anticipation.

Overall, PT toward avatars is able to influence SRC, which indicates a change in the mental representation of the situation, if the right conditions are met. As a result, PT can lead to an effect of spatial correspondence as seen from the avatar’s point of view rather than the person’s, effectively reversing the expected effect of spatial compatibility under certain circumstances (Böffel and Müsseler, 2017; Müsseler et al., 2017). Assuming that SRC effects arise based on the mental representation of a task, it is a useful tool to quantify PT because it allows us to infer how the stimulus location is coded (Ottoboni et al., 2005; Hommel, 2011). A tool that we also rely on in the present study.

Based on the described mechanisms, we expect that an increase in perceived ownership aids the incorporation of the avatar and its movements into the person’s mental representation of the task. This should lead to an increase of visual PT to facilitate action planning and therefore induce larger compatibility effects from the avatar’s perspective. We hope to show what is ultimately more beneficial: high ownership of the avatar or low ownership but higher levels of autonomy on the side of the avatar. We believe that although PT can be an effective mechanism for understanding someone else’s actions (Tversky and Hard, 2009) it is even more vital when it helps to plan our own actions through the means of an avatar. Or to put it differently: we think that planning our own actions evokes a stronger need for visual PT than understanding someone else’s.

To summarize the goals of this study: first, we want to show that the otherwise automatic acquisition of perceived ownership of an object can be influenced in a top–down manner by a framework provided in the instruction of the task. And second, we want to demonstrate that this change in perceived ownership is associated with changes in visual PT as measured with stimulus compatibility effects. Therefore, we want to pose the following hypotheses.

### Hypotheses

We expect that the two different instructions produce quantifiable differences in perceived ownership of the avatar, measured by the avatar-ownership questionnaire with higher self-reported perceived ownership in the high-ownership group in comparison to the low ownership group. We further predict that SRC effects are dependent on perceived ownership. In the high ownership group, we expect a larger benefit of spatially non-corresponding conditions compared to the low ownership group where compatibility drifts toward the participant’s perspective rather than the avatar’s. This should result in an interaction of spatial correspondence and instruction.

## Materials and Methods

**FIGURE 1:**
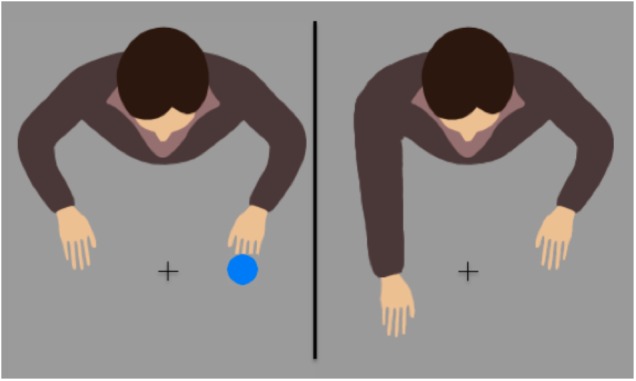
Example condition that demanded a contralateral response, before **(left)** or after **(right)** a right key-press.

We used two different instructions for the same task to top–down influence perceived ownership of an avatar: the setup and the avatars used were similar to the ones of Böffel and Müsseler (2017) and Müsseler et al. (2017). The participants were confronted with an avatar that was displayed on a screen and sitting opposite them (**Figure 1**). One instruction described the avatar as fully controlled by the participant, much like a tool (high ownership condition), and the second tried to establish the avatar as an individual agent (low ownership condition). The participants were asked to respond to dark/light blue disks with key presses that resulted in avatar hand movements. In both groups, stimuli, responses, and action effects were identical. The action goal was defined in the same way in both groups: act so that the avatar moves a certain arm. This was done to avoid the influence of different action goals that could otherwise lead to SRC effects related to the location of the action goal rather than response location as described by Hommel (1993a).

### Participants

In total 48 students (39 females) from RWTH Aachen University with a mean age of *M* = 21.6 (*SD* = 3.9) participated in this experiment for course credit or a monetary compensation of 5 €. All participants had normal or corrected-to-normal vision.

### Apparatus and Stimuli

**FIGURE 2:**
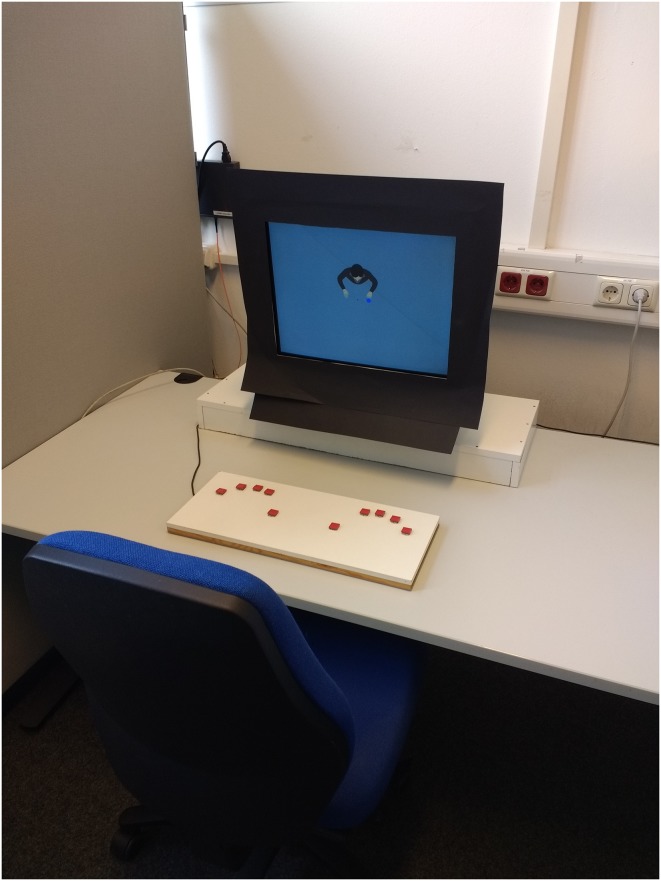
Experimental setup.

MatLab and the Psychtoolbox Extension v3.0 (Brainard, 1997; Pelli, 1997) were used for stimulus presentation and reaction time measurement. The stimuli were presented on a 22″ CRT monitor (Iiyama Visionmaster Pro 514 with a resolution of 1024 × 768 and 100 Hz refresh rate). The participants were seated 70 cm in front of the monitor and responded with their left and right index fingers on response keys (**Figure 2**). Dark blue (RGB 36 115 254) and light blue circles (RGB 98 193 254), each with a diameter of 50 pixel (1.79°) were used as targets, presented 1.61° to the left or right of a central fixation cross and in front of a gray background (RGB 155 155 155). The avatar had a size of roughly 240 × 200 pixels (8.73° × 8.56°) and was facing the participants with its hands pointing toward the stimuli positions (**Figure 1**).

### Procedure

The participants gave written informed consent to the terms of the experiment, including data storage and data usage for publication purposes. After that, half of the participants were instructed to control the avatar’s hands by pressing the respective key on the response board: a right key-press moves the right hand and a left key-press moves the left hand. This lead to effector congruency between the participant’s hands and the hands of the avatar. The second group was instructed to imagine the avatar as an independent agent that acts according to its own goals and always wants to move both hands. However, the participant can prevent the avatar from moving the ipsilateral hand with a key press. For example, a right key-press stops the avatar’s left hand from moving. As a result, the avatar once again only moves its right hand. This means that in both groups the same key press lead to the same observable action effects and the main objective was the same in both instructions: act in such a way that only the contralateral hand is moved if the target is light-blue and only the ipsilateral hand is moved if the target is dark-blue. The mapping of light-and dark-blue targets to ipsi- and contralateral responses was counterbalanced between participants. Because the avatar always showed the same hand movements after a certain key is pressed, regardless of the instruction used, and the goal of the action was always the same only the interpretation of the situation was changed by the instruction. A central fixation cross and the avatar remained visible throughout the experiment. The targets were presented without a time limit until the participants responded. If the response was incorrect, slower than 1,500 ms (lapse) or faster than 100 ms (anticipation) it was labeled as an error and followed by a feedback tone. The waiting period between the response and the beginning of the next trial was 2,250 ms and increased by additional 1,500 ms after an error occurred. Each participant performed 10 blocks, including 8 repetitions of each combination of stimulus position and stimulus color. The first block was a practice block that was excluded from the analysis. The order of trials was randomized within each block. Overall each condition was repeated 80 times over the course of the experiment resulting in a total of 320 trials per participant, excluding practice-trials. The participants needed approximately 25 min to complete the experiment.

After the experiment the participants were asked to fill in a questionnaire that featured the perception of the avatar. The avatar-questionnaire was based on an instrument used by Ma and Hommel (2015b) that targeted the perceived ownership of virtual hands and is a modified version of the questionnaire Botvinick and Cohen (1998) used to examine the rubber hand illusion. Our modified questionnaire asked the participants to rate 10 statements regarding ownership of the avatar and its hands (e.g., “It felt as if the avatar’s hands were part of my body” “The hands of the avatar began to resemble my hands in terms of shape or skin tone”) on a seven-step Likert scale ranging from 1 “I strongly disagree” to 7 “I strongly agree.” The complete list of items used is shown in **Table 1**. We altered the items used by Ma and Hommel (2015b) to closer resemble the avatar setting while trying to maintain the general objective of the instrument. Three items were omitted because they targeted tactile perceptions, which were not included in our experiment. The instrument was used in a German translation. We calculated an overall perceived ownership score as the sum of the responses for each participant. The possible range of ownership values was therefore 10 to 70 and higher values indicated higher levels of perceived ownership.

**Table 1:** Items used in the ownership questionnaire.

Q1: It felt as if the avatar’s hands were part of my body.
Q2: It seemed that my hand was in the location where the hand of the avatar was.
Q3: I lost the feeling where my hands were located.
Q4: It seemed that my hands were no longer part of my body.
Q5: I had the feeling that I might have additional hands.
Q6: Sometimes I felt as if my hands were turning virtual.
Q7: The hands of the avatar began to resemble my hands in terms of shape or skin tone.
Q8: It appeared (visually) as if the hands of the avatar were drifting toward my hands.
Q9: It seemed like I could have moved the hand on the screen if I wanted, as if it were obeying my will.
Q10: It felt as if my hands took on the same size as the avatar’s hands.


*The items are based on the instruments used by Botvinick and Cohen (1998) and Ma and Hommel (2015b).*

### Design

The experimental conditions consisted of all possible combination of stimulus position, response position and instruction. Stimulus and response position were used to determine spatial correspondence. The conditions in which stimulus and response positions were both on the participants left or right were labeled as spatially corresponding, others as non-corresponding. This resulted in a 2 × 2 design with the within-subjects factor spatial correspondence (non-corresponding vs. corresponding) and the between-subjects factor instruction (high ownership vs. low ownership).

## Results

### Reported Ownership

The analysis of the avatar-questionnaire data revealed instruction-based group differences: the high-ownership instruction was associated with overall higher levels of self-reported ownership (*M* = 22.3; *SD* = 10.9) compared to the low ownership instruction (*M* = 17.8; *SD* = 7.0). This effect was statistically significant [*t*(39.15) = 1.69; *p*_(one-tailed)_ = 0.05], *df* were Welch-adjusted to account for differing variances in both groups.

### Reaction Times and Percentage Errors

**FIGURE 3:**
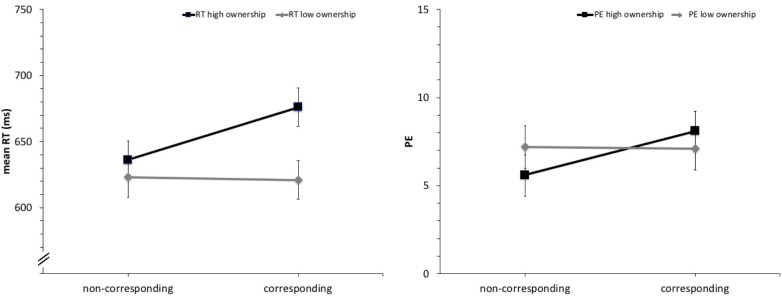
Mean reaction times (RTs) and percentage errors (PEs) as a function of spatial correspondence and instruction (high vs. low ownership). Error bars represent 95% within-subject CIs (Morey, 2008).

Reaction times longer than 1,500 ms or shorter than 100 ms were regarded as errors and were removed from the RT analyses. A total of 254 trials (1.7%) were excluded this way along with 821 false responses (5.3%) for a total of 1075 errors (7.0%). Mean RTs and percentage errors (PE) were analyzed separately using 2 × 2 mixed design ANOVAs with the within-subjects factor spatial correspondence (corresponding vs. non-corresponding) and the between subject factor instruction (high vs. low ownership). Results are shown in **Figure 3**. The analysis of mean reaction times revealed a significant influence of spatial correspondence *F*(1,46) = 5.51, *p* = 0.023, $\eta_{p}^{2}$ = 0.11, overall favoring spatially non-corresponding stimulus–response pairings (*M*_corr._ = 649 ms vs. *M*_non-corr._ = 630 ms). This effect was significantly influenced by the factor instruction *F*(1,46) = 7.04, *p* = 0.011, $\eta_{p}^{2}$ = 0.13 with a 40 ms advantage of non-corresponding conditions in the high ownership instruction group compared to a 2 ms advantage of spatially corresponding conditions in the low ownership group. Analyzed separately, the 40 ms advantage of non-corresponding conditions in the high ownership condition is statistically significant with [*t*(23) = 3.69, *p* = 0.001, two tailed] while the 2 ms advantage of spatially corresponding conditions in the low ownership group is not [*t*(13) = 0.20, *p* = 0.84, two tailed].

The analysis of percentage errors showed a marginally significant main effect of spatial correspondence *F*(1,46) = 3.58, *p* = 0.065, $\eta_{p}^{2}$ = 0.07 that interacted significantly with instruction *F*(1,46) = 4.40, *p* = 0.041, $\eta_{p}^{2}$ = 0.09. Spatially non-corresponding SR-mappings were associated with lower error rates compared to corresponding ones in the high-ownership instruction (*M*_corr._ = 8.1% vs. *M*_non-corr._ = 5.6%) but not when paired with the low ownership instruction (*M*_corr._ = 7.1% vs. *M*_non-corr._ = 7.2%). Similar to the reaction times only the 2.5% points advantage of non-corresponding conditions in the high ownership condition is statistically significant with [*t*(23) = 3.57, *p* = 0.002, two tailed] while the 0.1% points advantage of spatially corresponding conditions in the low ownership group is not [*t*(13) = 0.13, *p* = 0.90, two tailed] when analyzed separately.

## Discussion

The analysis of the avatar-questionnaire data revealed instruction-based group differences that were consistent with our expectations. The high ownership instruction resulted in significantly higher values of self-reported ownership compared to the low ownership instruction. We therefore conclude that the manipulation was successful. Overall this supports the idea that top–down processes influence perceived ownership.

The analysis of reaction times and error rates showed that both instruction cause different effects of spatial correspondence in otherwise identical scenarios. While the correspondence effect was negligible in the low ownership condition, it was significantly (more) negative in the high-ownership condition. The high ownership conditions therefore cause compatibility effects that are based on the avatar’s point of view instead of the participant’s own. This means that the observed effects are similar to the effects we would expect if the participant would actually see the scene from a rotated point of view. We think this is a very strong indicator that the stimuli are coded from the avatar’s viewpoint and that the resulting mental representation is the important factor that determines spatial compatibility rather than the actual physical location of the stimuli. As a result, stimuli presented on the left produced compatibility effects as if they were presented on the right and vice versa. A stimulus presented on the left side of the avatar lead to the formation of the same feature code as a stimulus presented on the left of the person, even though their position is in fact different. This is apparently not the case in the low ownership condition which indicates that both conditions lead to different mental representation of the same scene.

The absence of a correspondence effect in the low ownership could point toward the possibility that the task was complicated enough to eliminate the influence of the automatic activation of spatially corresponding responses a phenomenon that can be observed in mixed SRC tasks (Shaffer, 1965). This is most likely a result of a reactive inhibition rather than proactive suppression of the automatic route in complex situations and was described by Proctor and Vu (2010). A similar case could be made for the low ownership condition in our experiment, because the instruction might be sufficiently complex to cause a similar effect. This is apparently not the case in the high ownership condition where an advantage of spatially non-corresponding conditions was observed. The analysis of reaction times showed higher mean reaction times in the high ownership group. Although this difference is not statistically significant (*p* = 0.17) it seems unlikely that the low-ownership condition is overall more complex.

Why do we still observe a correspondence effect and why is the automatic route not inhibited in the high ownership group? The high-ownership condition has the advantage that the task can be broken down into several steps: step 1: perspective taking, step 2: recoding of the stimulus position, step 3: action. While each step is relatively simple, the completion of all steps combined might cause higher reaction times. The PT in step 1 is also associated with costs that would explain the numerically higher reaction time compared to the low ownership group (Janczyk, 2013). We propose that after PT is completed, the spatial information of the stimulus would be coded within the new frame of reference from the avatar’s point of view. At this point the task is identical to a typical SR compatibility task. It leads to the expected effects when accounting for the new mental representation of the stimulus. This mechanism could be similar or identical to the concept of referential coding (Hommel, 1993b) that lays the groundwork for the coding of stimulus features based on different reference frames. The new reference frame provided by the avatar would be rotated by 180° from the participant’s point of view and constitutes a rather drastic example of conflicting reference frames. This supports the theory that referential coding of the same situation can either be based on an egocentric or alternative reference frame, based on expectations and knowledge about the situation.

An alternative explanation for the absence of compatibility effects in the low ownership condition might be that both reference frames are activated equally strong, leading to the stimulus position being coded as neutral. The stimulus would cause the formation of both feature codes: “left” and “right.” This conflict might result in an overall compatibility effect of zero. Alternatively, one frame of reference may always overwrite the other but both reference frames win this conflict equally often, resulting in a zero-sum of spatial correspondence effects. The participant might therefore switch between both reference frames, but only one of them would be active at a given time. If the latter is the case, the automatic route of the dual-route model might still be active but its effects are evened-out over the course of the experiment. An alternating activation of the egocentric and allocentric reference frame within the same condition could effectively cause further mixing of compatible and incompatible mappings within those conditions and cause an elimination of SRC effects as described earlier (Shaffer, 1965). It is also possible that the low ownership instruction was interpreted differently by different individuals, leading to PT and reversed spatial correspondence effects in some, but classic correspondence effects in others, again evening out.

Overall this study provides evidence for the influence of top–down processes in perceived ownership, but to conclude that bottom–up processes are not important in the present situation might be a mistake. In this experiment the situation included a reliable congruency between the participant’s responses and the movement of the avatar. Such characteristics are expected to invoke a sense of ownership of the avatar (Ma and Hommel, 2015b) that should require no further explanation or instruction. Based on the results of the present study it seems more likely that top–down processes can suppress perceived ownership of an avatar even if the situation would otherwise induce it.

Although we tried to ensure that the final action goal was the same in both instructions, we ultimately cannot rule the influence of sub-goals out. The imagined grabbing of the avatars ipsilateral hand is the most likely example of such a sub-goal. While the final intention is always to produce an avatar movement that is contralateral to the key press, this sub-goal would have an ipsilateral location of intention, in this case the prevention of a movement. A conflict of goal and sub-goal location could be a contributor to the absence of spatial correspondence effects in the low ownership group. How this prevention of an action effect as an intention influences action planning is not entirely clear, although there is some evidence that the location of intention is more important than the location of the actual effect (Hommel, 1993a; Müsseler et al., 2012).

There are some limitations of this study that are noteworthy. First, measuring ownership with a self-report questionnaire might not be ideal. It is unclear whether participants are able to consciously perceive ownership of the same magnitude as it actually influences their actions. On one hand, it might very well be possible that the artificial setting in this study prevents higher degrees of reported ownership, because it seems difficult to agree to the items used in the questionnaire and the process could be largely subconscious. As a result, true ownership effects might be underreported. On the other hand, social desirability bias might have caused an overestimation of ownership. As a result, other means of measuring ownership might be more feasible than self-report. The second limitation lies in the abstract nature of the avatars used in this experiment. The avatars offered no customization and were the same for each participant. While this design-choice ensured constant conditions for all participants, it might have had negative impact on perceived ownership. A higher degree of physical similarity between participant and avatar might result in higher ownership and stronger effects. Customization is a feature that is often, although not always, present in applications that use avatars. Whether the tradeoff of visual constancy for all participants at the cost of higher variability in similarity between participant and avatar is justified is up for debate. From a psychophysics point of view, constancy is crucial whereas from an applied perspective it is often negligible. Since this study relies on a SR compatibility task — a classic experimental paradigm — we chose the first option. Last but not least, it is also likely that the two instructions not only influenced perceived ownership but also the social character of the situation. With high ownership, the situation might be perceived as less social and closer to a tool-use scenario compared to the low ownership instruction that established the avatar as an independent agent. This is particularly interesting, since PT is often seen as a social phenomenon, yet in this study it is only measurable in the situation that is effectively less social.

The result of this study can be applied in the design of human–computer interactions (HCIs). When the user is required to act from the perspective of an avatar in the presence of other avatars, establishing those distracting avatars as independent agents could prove useful to prevent PT toward these distractors. Such unwanted PT could create additional reference frames that are potentially associated with costs and conflicting SRC relations. On the other hand, stressing the control over an avatar might facilitate PT toward this avatar.

## Data Availability

The raw data supporting the conclusions of this manuscript will be made available by the authors, without undue reservation, to any qualified researcher.

## Ethics Statement

In accordance with the Declaration of Helsinki (World Medical Association, 2013), all participants gave written informed consent to participate in the study and participation was voluntary. Further, no undue physical or psychological stress by participating in this study was anticipated and the data obtained on individual participants were not used to elucidate properties of the participant but to examine general laws of cognitive information processing. As a result, no ethical concerns were identified in accordance to the ethics guidelines of the DFG (Deutsche Forschungsgemeinschaft [German Research Foundation], 2009).

## Author Contributions

CB developed the study concept and design, performed the data collection and data analysis under the supervision of JM. All authors have approved this version of the manuscript and its submission.

## Conflict of Interest Statement

The authors declare that the research was conducted in the absence of any commercial or financial relationships that could be construed as a potential conflict of interest.
